# Analysis of treatment outcomes according to the cycles of adjuvant chemotherapy in gastric cancer: a retrospective nationwide cohort study

**DOI:** 10.1186/s12885-022-10006-7

**Published:** 2022-09-03

**Authors:** Tae-Hwan Kim, Mi Sun Ahn, Yong Won Choi, Seok Yun Kang, Jin-Hyuk Choi, Hyun Woo Lee, Minae Park, Hasung Kim

**Affiliations:** 1 Department of Hematology-Oncology, Ajou University School of Medicine, 164 World cup-ro, Yeongtong-gu, Suwon, 16499 Gyeonggi-do Korea; 2 Data Science Team, Hanmi Pharm. Co. Ltd, Seoul, Korea

**Keywords:** Adjuvant, Chemotherapy, Gastric cancer, Survival, Nationwide cohort study

## Abstract

**Background:**

One-year S-1 or six-month capecitabine/oxaliplatin (CAPOX) has been the standard adjuvant chemotherapy for gastric cancer (GC). We investigated outcomes according to the cycles of adjuvant chemotherapy, using data from the Korean Health Insurance and Assessment Service.

**Methods:**

A total of 20,552 patients, including 13,614 patients who received S-1 and 6,938 patients who received CAPOX extracted from 558,442 patients were retrospectively analyzed. The five-year overall survival rate was evaluated according to the duration of adjuvant chemotherapy.

**Results:**

The five-year overall survival rate gradually increased according to the increase in adjuvant chemotherapy cycles in both the S-1 (≤ 5 cycles: 48.4%, hazard ratio [HR] 4.06, 95% confidence interval [CI] 3.74–4.40, *P* < 0.0001; 5 < cycles ≤ 6: 55.4%, HR 3.08, 95% CI 2.65–3.57, *P* < 0.0001; 6 < cycles ≤ 7: 64.1%, HR 2.11, 95% CI 1.84–2.41, *P* < 0.0001; 7 < cycles < 8: 71.1%, HR 1.60, 95% CI 1.39–1.84, *P* < 0.0001; ≥ 8 cycles: 77.9%) and the CAPOX groups (≤ 4 cycles: 43.5%, HR 3.20, 95% CI 2.84–3.61, *P* < 0.0001; 5 cycles: 45.3%, HR 2.63, 95% CI 2.11–3.27, *P* < 0.0001; 6 cycles: 47.1%, HR 2.09, 95% CI 1.76–2.49, *P* < 0.0001; 7 cycles: 55.3%, HR 1.63, 95% CI 1.35–1.96, *P* < 0.0001; ≥ 8 cycles: 67.2%).

**Conclusions:**

Reducing the treatment cycles of adjuvant chemotherapy in GC with S-1 or CAPOX showed inferior survival outcomes. Completing the standard duration of adjuvant chemotherapy with S-1 or CAPOX would be strongly recommended.

## Background

Gastric cancer (GC) is the most common newly diagnosed malignancy in Korea and the fourth most common malignancy worldwide [1, 2]. Although the clinical significance of neoadjuvant chemotherapy for the treatment of locally advanced GC has recently emerged, the benefits of neoadjuvant chemotherapy has shown conflicting results depending on the proportion of patients who underwent D2 lymphadenectomy in each study, therefore, adjuvant chemotherapy after gastrectomy with D2 lymphadenectomy has been the mainstay of standard treatment [3–7].

S-1 is an oral fluoropyrimidine derivative used for chemotherapy in various gastrointestinal malignancies [8]. After the results of the Adjuvant Chemotherapy Trial of S-1 for Gastric Cancer (ACTS-GC) study were published, one-year adjuvant treatment of S-1 for GC was established as a standard treatment [9, 10]. In addition, the results of the Capecitabine and Oxaliplatin Adjuvant Study in Stomach Cancer (CLASSIC) trial proved the effectiveness of an adjuvant chemotherapeutic regimen with capecitabine (i.e., an oral fluoropyrimidine carbamate) [11], and six-month therapy of capecitabine/oxaliplatin (CAPOX) has also been used as a standard treatment for GC [12].

Furthermore, a study on shortening the duration of adjuvant S-1 failed to show the noninferiority of survival outcome for six-month adjuvant chemotherapy of S-1 compared with the one-year standard treatment [9, 13].

Therefore, we aimed to examine the survival outcomes according to the duration and numbers of cycles of adjuvant chemotherapy, using the data of a large population from the Korean Health Insurance Review and Assessment Service (HIRA).

## Methods

### Patients

**Fig. 1:**
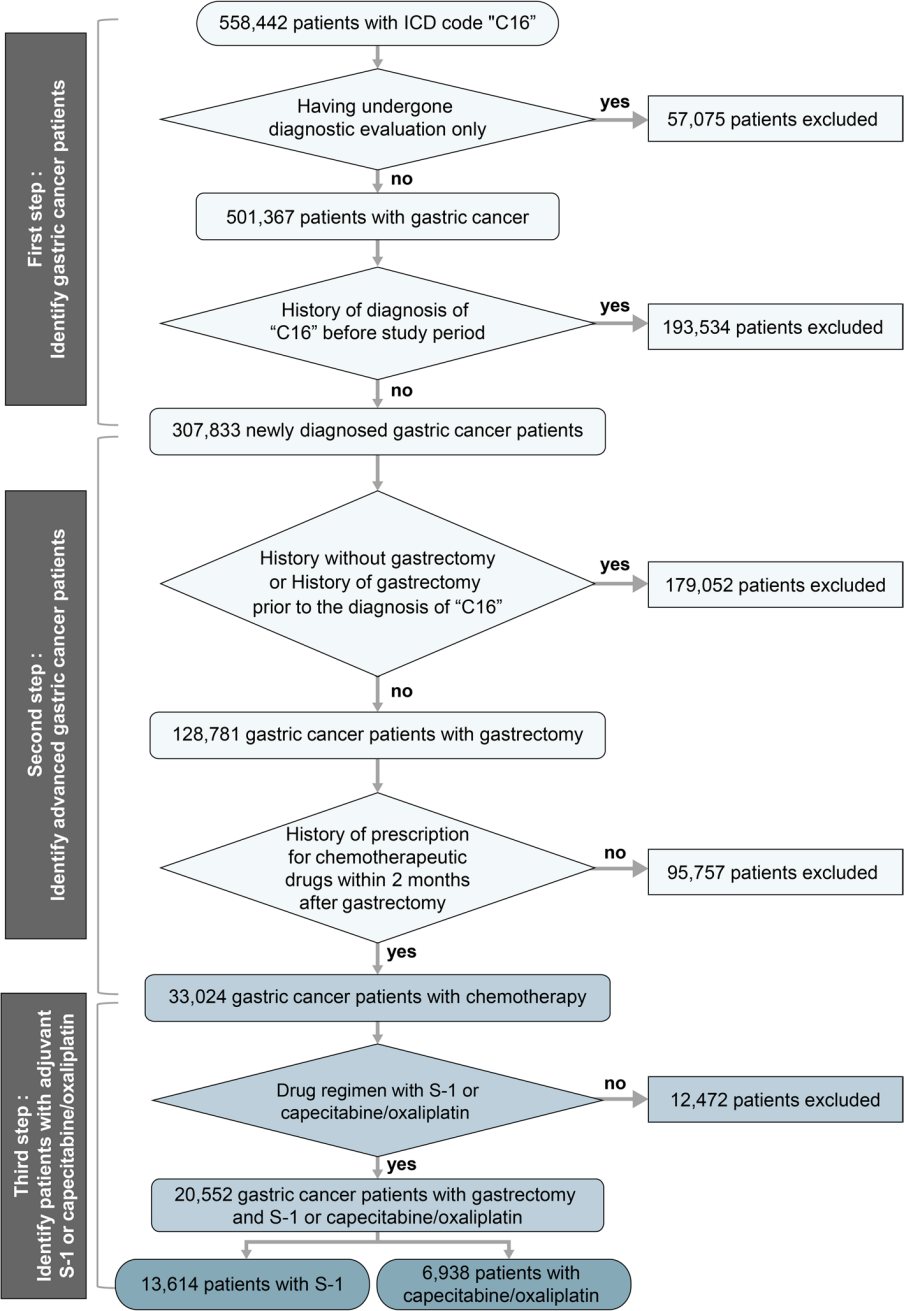
Study design. ICD indicates the International Classification of Diseases.

A total of 558,442 patients were identified with the C16 code from the International Classification of Diseases in the HIRA data during the study period of January 1, 2011 until December 31, 2018. The patients who had only undergone diagnostic evaluation without treatment were excluded, which left 501,367 patients. In addition, 193,534 patients with a history of a C16 code diagnosis before the study period and 179,052 patients with no history of gastrectomy or who had a history of gastrectomy prior to the diagnosis with the C16 code were also excluded. Among the remaining 128,781 patients, we analyzed for 33,024 patients who were prescribed chemotherapeutic drugs within two months after surgery. Of these, 20,552 patients who were treated with the chemotherapeutic drug regimen of S-1 or CAPOX, which are reimbursable drugs in the Korean health insurance system, were the subjects of the final analysis, excluding the patients treated with other chemotherapeutic drugs or combination regimens with S-1 or CAPOX (Fig. 1).

The research protocol was approved by the institutional review board (IRB) of Ajou University Hospital (IRB approval no. AJIRB-MED-EXP-18–489). Informed consent was waived by the IRB because this study was conducted using the medical records of anonymized patients.

### Clinical review and definition of survival outcomes

The baseline patient characteristics identified using the HIRA data were age, gender, and comorbidities, including diabetes mellitus, hypertension, chronic obstructive pulmonary disease, and dyslipidemia. Because one S-1 cycle comprises a four-week treatment and a two-week rest, the S-1 cycles were identified for the prescribed days [9]. In addition, the numbers of prescriptions of oxaliplatin were confirmed for the identification of cycles in the CAPOX group.

A patient death was operationally defined as an event of follow-up loss with no clinical records or drug prescriptions for more than six months [14], because the exact date of death could not be identified using the HIRA data, furthermore, the date of death was defined as the date of the patient’s last medical record. The five-year overall survival (OS) rates were investigated from the start date of chemotherapy, while data on the survivors were censored on December 31, 2018.

### Statistical analysis

The baseline characteristics according to the adjuvant chemotherapeutic regimen were compared using the Yate’s chi-squared test. The five-year OS rates were calculated using the Kaplan–Meier method. The Cox proportional hazard model was used to analyze the differences between the survival curves according to the duration of adjuvant chemotherapy. All statistical analyses were two-sided and performed using SAS software, version 9.4 (SAS Institute, Cary, NC, USA).

## Results

### Patient characteristics

A total of 20,552 patients were analyzed: 13,614 patients who received S-1 and 6,938 patients who received CAPOX. Of these, 4,676 patients (S-1: 3,137 patients, CAPOX: 1,539 patients) were concluded to have died according to the operational definition. The most common durations of follow-up loss for patients defined as having died were 1–2 years (S-1: 746 patients [23.8%]; CAPOX: 411 patients [26.7%]) and 2–3 years (S-1: 554 patients [17.7%]; CAPOX: 347 patients [22.5%]). The numbers of the patients with duration of follow-up loss between six months and one year were 591 patients (18.8%) for S-1 and 301 patients (19.6%) for CAPOX, respectively.

The patient characteristics are summarized in Table 1. The mean age was 61.4 years, and patients aged in their 50 s, 60 s and 70 s accounted for the largest proportions: 25.5%, 28.5%, and 24.9%, respectively. Male patients predominated (*N* = 14,063; 68.4%).

**Table 1:** Patient characteristics

Clinical characteristics	Total (*N* = 20,552)	S-1 (*N* = 13,614)	Capecitabine/oxaliplatin (*N* = 6,938)	*P* Value
Age, years, mean (SD)	61.4 (12.0)	63.3 (12.1)	57.6 (11.0)	< 0.0001
Age group, years, *N* (%)				
< 30	101 (0.5)	53 (0.4)	48 (0.7)	< 0.0001
30–39	826 (4.0)	447 (3.3)	379 (5.5)	
40–49	2,541 (12.4)	1,417 (10.4)	1,124 (16.2)	
50–59	5,231 (25.5)	2,980 (21.9)	2,251 (32.4)	
60–69	5,866 (28.5)	3,759 (27.6)	2,107 (30.4)	
70–79	5,126 (24.9)	4,166 (30.6)	960 (13.8)	
≥ 80	861 (4.2)	792 (5.8)	69 (1.0)	
Gender, *N* (%)				
Male	14,063 (68.4)	9,226 (67.8)	4,837 (69.7)	0.0047
Female	6,489 (31.6)	4,388 (32.2)	2,101 (30.3)	
Comorbidities, *N* (%)				
DM	4,772 (23.2)	3,360 (24.7)	1,412 (20.4)	< 0.0001
Hypertension	8,133 (39.6)	5,803 (42.6)	2,330 (33.6)	< 0.0001
Dyslipidemia	2,143 (10.4)	1,561 (11.5)	582 (8.4)	< 0.0001
COPD	6,712 (32.7)	4,535 (33.3)	2,177 (31.4)	0.0055

*Abbreviations*: *SD* Standard deviation, *DM* Diabetes mellitus, *COPD* Chronic obstructive pulmonary disease

The numbers of patients with diabetes mellitus, hypertension, dyslipidemia, or chronic obstructive pulmonary disease were 4,772 (23.2%), 8,133 (39.6%), 2,143 (10.4%), or 6,712 (32.7%), respectively. All comorbidities were significantly more common in the S-1 group (Table 1).

The patients who completed eight cycles of adjuvant chemotherapy were most common in both the S-1 and CAPOX groups (S-1: 50.9%; CAPOX: 60.0%). The patients who received S-1 for five cycles or fewer and CAPOX for four cycles or fewer were second most common (S-1: 28.7%; CAPOX: 20.4%). The proportions of patients treated with other cycles of adjuvant chemotherapy are shown in Table 2 and Table 3.

**Table 2:** Univariate and multivariate analyses about survival outcomes in the patients treated with S-1

**Characteristics**		**Number of patients (%)**	**Unadjusted HR (95% CI)**	***P* Value**	**Adjusted HR (95% CI)**	***P* Value**
	Total	13,614 (100.0)				
Age, years	< 30	53 (0.4)	1		1	
	30–39	447 (3.3)	0.71 (0.41–1.21)	0.207	0.69 (0.40–1.18)	0.172
	40–49	1,417 (10.4)	0.54 (0.32–0.91)	0.021	0.53 (0.31–0.89)	0.016
	50–59	2,980 (21.9)	0.61 (0.36–1.01)	0.057	0.57 (0.34–0.95)	0.032
	60–69	3,759 (27.6)	0.76 (0.46–1.27)	0.294	0.70 (0.42–1.17)	0.173
	70–79	4,166 (30.6)	1.18 (0.71–1.96)	0.528	0.95 (0.57–1.58)	0.834
	≥ 80	792 (5.8)	2.10 (1.25–3.53)	0.005	1.44 (0.85–2.43)	0.175
Gender	Male	9,226 (67.8)	1		1	
	Female	4,388 (32.2)	0.86 (0.80–0.93)	0.0002	0.83 (0.77–0.90)	< 0.0001
Comorbidities						
DM	No	10,254 (75.3)	1		1	
	Yes	3,360 (24.7)	1.25 (1.16–1.36)	< 0.0001	1.11 (1.01–1.21)	0.03
Hypertension	No	7,811 (57.4)	1		1	
	Yes	5,803 (42.6)	1.16 (1.08–1.25)	< 0.0001	0.89 (0.82–0.96)	0.004
Dyslipidemia	No	9,079 (66.7)	1		1	
	Yes	4,535 (33.3)	1.04 (0.97–1.13)	0.292	0.96 (0.88–1.05)	0.375
COPD	No	12,053 (88.5)	1		1	
	Yes	1,561 (11.5)	1.19 (1.07–1.33)	0.002	0.99 (0.89–1.10)	0.839
Chemotherapy cycles	≥ 8 cycles	6,930 (50.9)	1		1	
	7 < cycles < 8	1,071 (7.9)	1.60 (1.39–1.84)	< 0.0001	1.54 (1.34–1.78)	< 0.0001
	6 < cycles ≤ 7	990 (7.3)	2.11 (1.84–2.41)	< 0.0001	2.03 (1.78–2.32)	< 0.0001
	5 < cycles ≤ 6	709 (5.2)	3.08 (2.65–3.57)	< 0.0001	2.86 (2.47–3.32)	< 0.0001
	≤ 5 cycles	3,914 (28.7)	4.06 (3.74–4.40)	< 0.0001	3.64 (3.35–3.95)	< 0.0001

*Abbreviations*: *HR* Hazard ratio, *CI* Confidence interval, *DM* Diabetes mellitus, *COPD* Chronic obstructive pulmonary disease

**Table 3:** Univariate and multivariate analyses about survival outcomes in the patients treated with capecitabine/oxaliplatin

**Characteristics**		**Number of patients (%)**	**Unadjusted HR (95% CI)**	***P* Value**	**Adjusted HR (95% CI)**	***P* Value**
	Total	6,938 (100.0)				
Age, years	< 30	48 (0.7)	1		1	
	30–39	379 (5.5)	0.77 (0.45–1.33)	0.353	0.89 (0.52–1.54)	0.680
	40–49	1,124 (16.2)	0.77 (0.46–1.30)	0.324	0.91 (0.54–1.53)	0.724
	50–59	2,251 (32.4)	0.69 (0.41–1.15)	0.156	0.81 (0.49–1.36)	0.431
	60–69	2,107 (30.4)	0.71 (0.43–1.19)	0.197	0.83 (0.50–1.40)	0.491
	70–79	960 (13.8)	0.88 (0.52–1.49)	0.637	0.92 (0.54–1.56)	0.764
	≥ 80	69 (1.0)	1.57 (0.82–3.01)	0.174	1.37 (0.71–2.66)	0.346
Gender	Male	4,837 (69.7)	1		1	
	Female	2,101 (30.3)	1.05 (0.94–1.17)	0.38	1.00 (0.90–1.12)	0.992
Comorbidities						
DM	No	5,526 (79.6)	1		1	
	Yes	1,412 (20.4)	1.01 (0.90–1.15)	0.828	1.02 (0.89–1.17)	0.767
Hypertension	No	4,608 (66.4)	1		1	
	Yes	2,330 (33.6)	0.94 (0.85–1.05)	0.271	0.92 (0.81–1.04)	0.183
Dyslipidemia	No	4,761 (68.6)	1		1	
	Yes	2,177 (31.4)	0.95 (0.85–1.07)	0.398	0.96 (0.84–1.08)	0.471
COPD	No	6,356 (91.6)	1		1	
	Yes	582 (8.4)	1.06 (0.88–1.27)	0.535	0.97 (0.80–1.16)	0.702
Chemotherapy cycles	≥ 8 cycles	4,161 (59.9)	1		1	
	7 < cycles < 8	514 (7.4)	1.63 (1.35–1.96)	< 0.0001	1.63 (1.35–1.97)	< 0.0001
	6 < cycles ≤ 7	531 (7.7)	2.09 (1.76–2.49)	< 0.0001	2.10 (1.76–2.49)	< 0.0001
	5 < cycles ≤ 6	317 (4.6)	2.63 (2.11–3.27)	< 0.0001	2.61 (2.10–3.26)	< 0.0001
	≤ 5 cycles	1,415 (20.4)	3.20 (2.84–3.61)	< 0.0001	3.16 (2.79–3.57)	< 0.0001

*Abbreviations*: *HR* Hazard ratio, *CI* Confidence interval, *DM* Diabetes mellitus, *COPD* Chronic obstructive pulmonary disease

### Patient outcomes

**Fig. 2:**
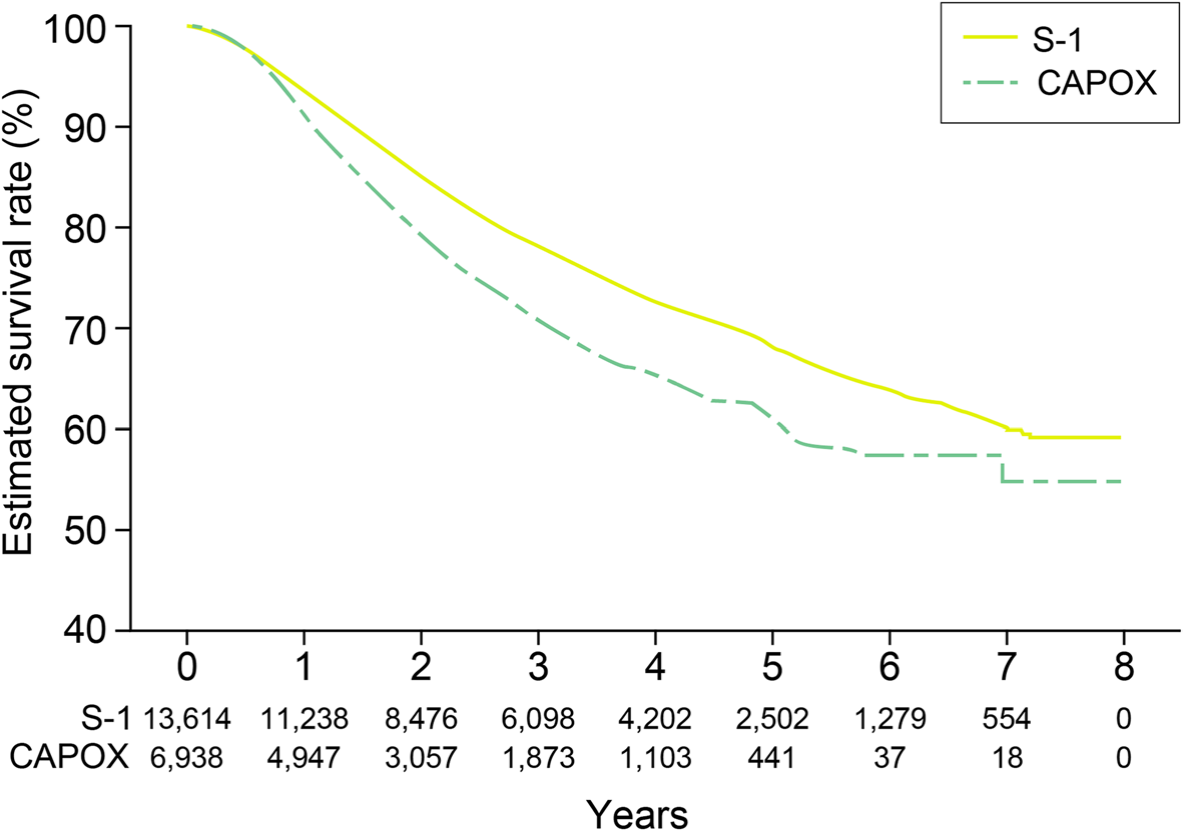
Overall survival rates according to the adjuvant chemotherapeutic regimen. CAPOX indicates capecitabine/oxaliplatin.

With a median follow-up duration of 2.3 years, the five-year OS rates were 68.2% for the patients treated with S-1 and 60.9% for the patients treated with CAPOX (Fig. 2).

**Fig. 3:**
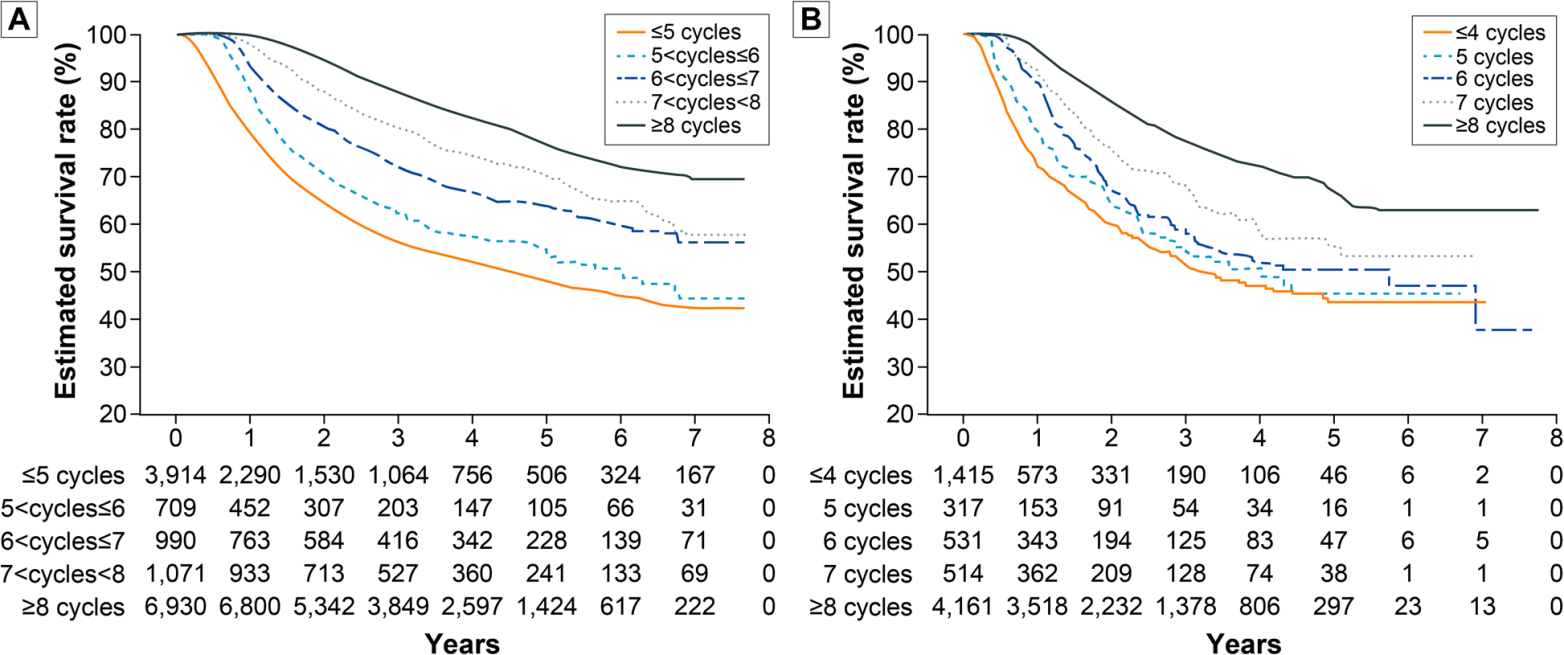
Overall survival rates of the patients with S-1 (**A**) and capecitabine/oxaliplatin (**B**) according to cycles

Both the S-1 and CAPOX groups showed statistically significant increases in five-year OS rates as the number of cycles of adjuvant chemotherapy increased. In the patients who received S-1, the five-year OS rates gradually increased from 48.4% to 55.4%, 64.1%, 71.1%, and 77.9% as the number of adjuvant chemotherapy cycles increased from five cycles or fewer to eight cycles or more, respectively (*P* < 0.0001). In addition, the same trend was identified in the patients with CAPOX from four cycles or fewer to eight cycles or more: 43.5%, 45.3%, 47.1%, 55.3%, and 67.2%, respectively (*P* < 0.0001) (Tables 2, and 3, and Fig. 3).

## Discussion

GC is one of the most common causes of cancer-related mortality worldwide [15, 16]. Since the results of the ACTS-GC and CLASSIC trials were published, one-year S-1 and six-month CAPOX adjuvant chemotherapy have been widely used in real-world practice. Surgery followed by adjuvant chemotherapy has generally been the standard treatment for locally advanced GC in Korea, because D2 lymph node dissection has been considered the standard procedure in East Asia, in contrast to Western Europe [17–20]. Therefore, those chemotherapeutic regimens have been reimbursable in the Korean national health insurance system, and we investigated the clinical outcomes of the adjuvant regimens in this real-world big data analysis.

In this study, the five-year OS rates for the patients who received CAPOX were poorer than those for the patients who were treated with S-1. We assumed that there were more patients with stage III GC in the CAPOX group because the S-1 adjuvant chemotherapy patients showed relatively poor survival outcomes for stage III [9, 21]. Although this study was a big data analysis, making it difficult to know the exact stage of the patients, it is estimated that the patients who received adjuvant treatment of CAPOX had a higher proportion of more advanced disease.

Our results showed that both the S-1 and the CAPOX groups had significantly better five-year OS rates as the number of cycles of adjuvant chemotherapy increased. A previous study was conducted to reduce the duration of adjuvant chemotherapy with a six-month S-1 regimen. However, that study showed poorer survival outcomes compared to the standard one-year treatment with S-1 [13]. Using a big data analysis of a larger populations in the real world, the current study showed equivalent results to the previous study. As a result, we recommend the completion of adjuvant chemotherapy with one-year S-1 or six-month CAPOX for GC as possible.

This study has some limitations. First, this was a retrospective study, and the study results cannot be generalized. Therefore, prospective studies will be needed. Second, this study was a big data analysis, and OS was operationally defined, because it was impossible to accurately identify the patients’ dates of death. Third, it is possible that a very small number of GC patients with stage IV who were treated with S-1 or CAPOX as palliative chemotherapy after palliative gastrectomy were included due to a limitation of HIRA database that does not provide the information of disease stage, which would have acted as an obstacle to more accurate analysis. Fourth, death was operationally defined as an event of follow-up loss with no clinical records or drug prescriptions for more than six months, which was likely to include some live patients. However, the proportion of patients with no clinical records between six months and one year was less than 20%, and most of the patients were those with no clinical records for more than one year. Fifth, D1 or D2 dissection, exact pathologic stage of GC patients in this study, or cause of decrease in cycles of adjuvant chemotherapy for each patient could not be identified as a limitation of Korean HIRA database. Sixth, Nonetheless, the five-year OS rates of this study results were comparable to those of the ACTS-GC and CLASSIC trials, considering that the patients who did not complete the standard duration of adjuvant chemotherapy were included [9, 22]. Therefore, it could be considered that sufficient trends were reflected. Despite some limitations, this study has great significance in demonstrating the benefits of adjuvant chemotherapy of one-year S-1 and six-month CAPOX through a real-world big data analysis.

## Conclusion

Reducing the treatment cycles of adjuvant chemotherapy in GC with S-1 or CAPOX showed inferior survival outcomes in a real-world big data analysis and completing the standard duration of adjuvant chemotherapy with S-1 or CAPOX would be strongly recommended.

## Availability data and materials

The datasets generated and/or analyzed during the current study are not publicly available due to the confidentiality of the data of patient but are available from the corresponding author on reasonable request.

## Abbreviations

GC: Gastric cancer
ACTS-GC: Adjuvant Chemotherapy Trial of S-1 for Gastric Cancer
CLASSIC: Capecitabine and Oxaliplatin Adjuvant Study in Stomach Cancer
CAPOX: Capecitabine/oxaliplatin
HIRA: Health insurance review and assessment service
IRB: Institutional review board
OS: Overall survival
